# A Review on Microorganisms in Constructed Wetlands for Typical Pollutant Removal: Species, Function, and Diversity

**DOI:** 10.3389/fmicb.2022.845725

**Published:** 2022-04-05

**Authors:** Jianwu Wang, Yuannan Long, Guanlong Yu, Guoliang Wang, Zhenyu Zhou, Peiyuan Li, Yameng Zhang, Kai Yang, Shitao Wang

**Affiliations:** ^1^School of Hydraulic and Environmental Engineering, Changsha University of Science & Technology, Changsha, China; ^2^Key Laboratory of Dongting Lake Aquatic Eco-Environmental Control and Restoration of Hunan Province, Changsha, China; ^3^Engineering and Technical Center of Hunan Provincial Environmental Protection for River-Lake Dredging Pollution Control, Changsha, China

**Keywords:** constructed wetlands, functional microorganisms, microbial diversity, pollutant removal, wastewater treatment

## Abstract

Constructed wetlands (CWs) have been proven as a reliable alternative to traditional wastewater treatment technologies. Microorganisms in CWs, as an important component, play a key role in processes such as pollutant degradation and nutrient transformation. Therefore, an in-depth analysis of the community structure and diversity of microorganisms, especially for functional microorganisms, in CWs is important to understand its performance patterns and explore optimized strategies. With advances in molecular biotechnology, it is now possible to analyze and study microbial communities and species composition in complex environments. This review performed bibliometric analysis of microbial studies in CWs to evaluate research trends and identify the most studied pollutants. On this basis, the main functional microorganisms of CWs involved in the removal of these pollutants are summarized, and the effects of these pollutants on microbial diversity are investigated. The result showed that the main phylum involved in functional microorganisms in CWs include *Proteobacteria*, *Bacteroidetes*, *Actinobacteria* and *Firmicutes*. These functional microorganisms can remove pollutants from CWs by catalyzing chemical reactions, biodegradation, biosorption, and supporting plant growth, etc. Regarding microbial alpha diversity, heavy metals and high concentrations of nitrogen and phosphorus significantly reduce microbial richness and diversity, whereas antibiotics can cause large fluctuations in alpha diversity. Overall, this review can provide new ideas and directions for the research of microorganisms in CWs.

## Introduction

Constructed wetlands (CWs) are passive biological engineering systems that use natural processes for wastewater treatment (Chen et al., 2021b; Zheng et al., 2021a). They have been widely used since the 1960s because of their simple operation, ease of maintenance, low cost, and environmental friendliness, providing a viable alternative to traditional wastewater treatment technologies (Zhao et al., 2016; Zheng et al., 2021a,b). They are mainly composed of substrate, plants, and microorganisms that purifying wastewater through the interaction of physical, chemical, and biological processes (Zhao et al., 2020c). Previous studies have shown that CWs can remove most environmental pollutants, including COD, N, P (Lin et al., 2002; Li et al., 2013; Zhao et al., 2019), heavy metals (Zhang et al., 2021e), and antibiotics (Liu et al., 2019), as well as some increasingly emerging pollutants (e.g., pesticides, flame retardants and persistent organic pollutants; Rajan et al., 2019; Long et al., 2021; Vymazal et al., 2021). Consequently, CWs are widely used in the treatment of domestic sewage, industrial wastewater, mine drainage, land leachate, polluted lake water, effluent from the livestock industry and other wastewater (Lin et al., 2002; Zhao et al., 2020c).

In CWs, microorganisms play a key role in pollutants removal, such as the degradation of organic pollutants and the conversion of various nutrients (Wang et al., 2020d). They can even use antibiotics as their sole carbon source (Ricken et al., 2013; Bessa et al., 2017). Regarding heavy metal compounds, which are generally difficult to biodegrade, microorganisms can also remove them from wastewater through biosorption, bioaccumulation and speciation transformation (Si et al., 2019). In addition, microorganisms can improve the tolerance and removal efficiency of CWs to pollutants by enhancing phytoremediation (Syranidou et al., 2018; Vassallo et al., 2020). In this context, in order to further optimize CWs, it is necessary to investigate the functional microorganisms associated with pollutant removal.

In recent years, advances in molecular biotechnology have largely facilitated intensive studies of microbial community structure and diversity (Arroyo et al., 2013). The advent of methods such as 16S sequencing, metagenomics sequencing, and high-throughput sequencing technologies has not only allowed a more accurate assessment of microbial diversity but only the analysis of the relative abundances of different microbial species and of the overall community structure (Zhao et al., 2016; Sanchez, 2017). In CWs, the diversity of microbial communities and the richness of certain species are key factors for efficient wastewater treatment (Zhao et al., 2020c). Therefore, in addition to the need for a summary of functional microorganisms, there is also a great need to study and analyze the effects of different pollutants on microbial diversity.

In recent year, various types of bibliometric analysis have been applied in different fields (Zhao et al., 2020b; Chen et al., 2021a; Kilicaslan et al., 2021). As a quantitative analysis technique, bibliometrics can reveal the current status and trends of a given research field by studying the distribution, quantitative relationships, and changing relationships of literature and information (Mao et al., 2015, 2018b; Liang and Gong, 2017). Overall, bibliometrics is a highly effective method of summarization and analysis and has become a useful tool when dealing with large amounts of scientific data (Mao et al., 2018a). This can help us to study the current state of research and trends in the field of microorganisms in CWs.

In this context, the purpose of this review is to first analyze, *via* bibliometrics, microbial-related articles in the field of CWs and several typical pollutants that are closely associated with microorganisms. Subsequently, the main functional microorganisms associated with the removal of targeted pollutants in CWs are systematically summarized and the effects of these pollutants on the diversity of microbial communities in CWs are discussed. This review will help us to further understand and explore the mechanisms of pollutant removal by microorganisms in CWs and the effects of pollutants in wastewater on microorganisms.

## Bibliometrics

Here, the bibliometrics approach is divided into two main parts: plotting of the publication trends of articles related to CWs and microorganisms using the Origin 2021 software and analyzing the keywords found in the publications in terms of microorganisms using the VOSviewer software.

## Data Collection

Respective data were obtained from the Web of Science (WOS) Core Collection database. And this review only considered the Science Citation Index Expanded (SCI-Expanded). To fully study the changes in the number of publications over the years, only publications from 1900 (the earliest time point that that can be set in the online SCI-Expanded database) to December 2020 were considered.

A search for CWs with the keywords “constructed wetland^*^” or “artificial wetland^*^” or “man-made wetland^*^” or “treatment wetland^*^” or “engineered wetland^*^” or “reed bed^*^” yielded 9,628 documents (the starting year is 1991, as the earliest record of microbial publications is from this year). The keywords “bacteri^*^ OR microb^*^ OR microorganism^*^” were searched for microbial, yielding a total of 2,764 documents. The year of publication and the bibliographic information of these publications (including authors, titles, source publications, abstracts, and references cited) were exported for subsequent analysis.

## Bibliometric Analysis

**Figure 1:**
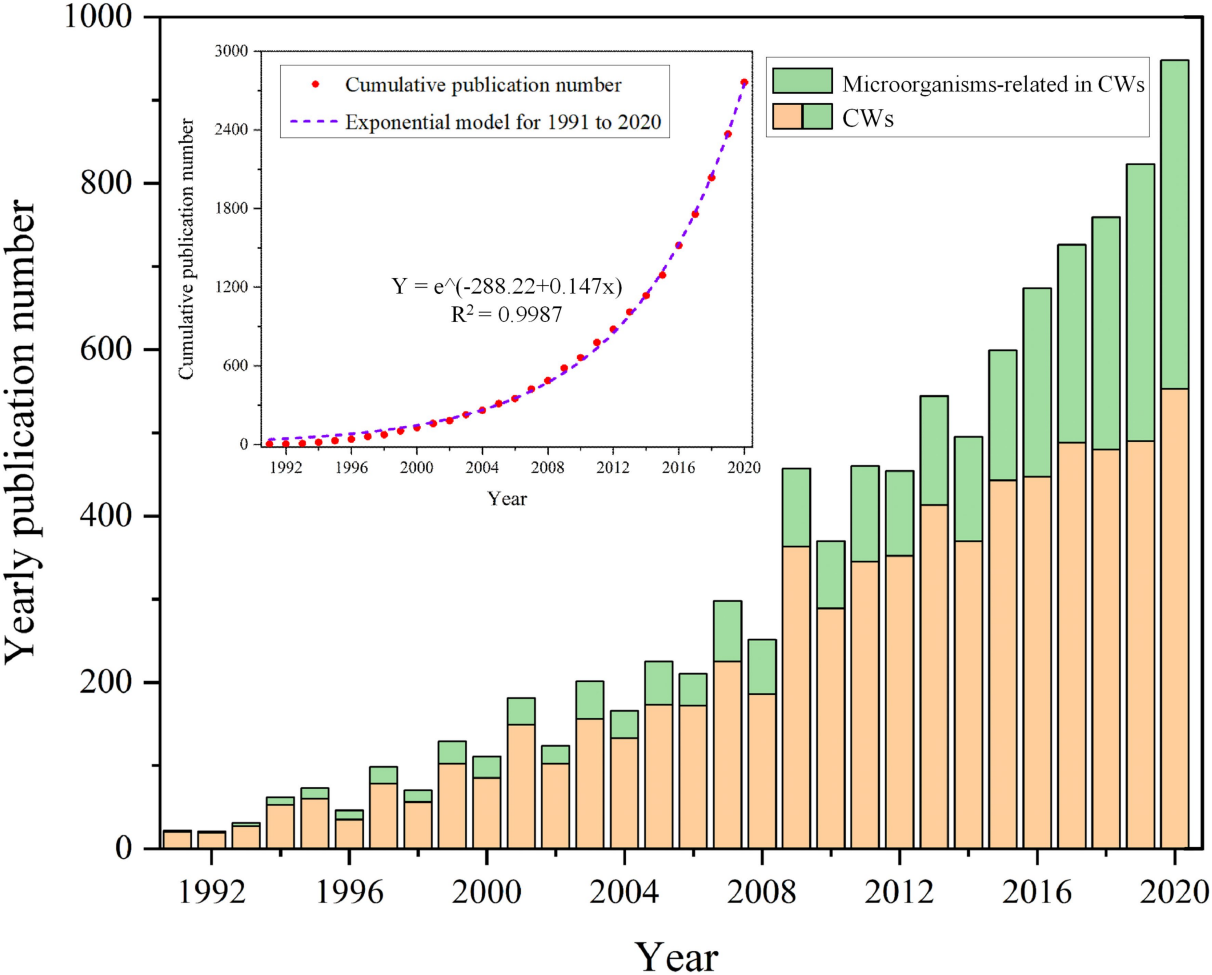
Yearly publications about CWs and microorganisms (cumulative publications curve about microorganisms).

The first aspect is the investigation of the general trend of microbial research by determining the change of articles involving microorganisms in CWs over time. For this, the yearly number of publications was counted and the articles about CWs and microorganisms were indicated separately by different colors to obtain the percentage of articles about microorganisms in the field of CWs (Figure 1). As can be seen in Figure 1, there is an overall upward trend in the number of yearly publications on CWs and microorganisms. Furthermore, an increasing number of publications involving CWs are related to microorganisms. These results reflect the wide use and study of CWs in an increasing number of countries as well as reflecting the increasing importance of microorganisms. Particularly in recent years with the advances in the molecular characterization of microbial communities, such as denaturing gradient gel electrophoresis or terminal fragment length polymorphism of PCR-amplified 16S ribosomal RNA gene fragments, as well as metagenomics, have greatly contributed to the development of microbial ecology research (Zhao et al., 2016; Sanchez, 2017; Rajan et al., 2019). This can be corroborated with the increasing trend of cumulative publication volume of microbial articles in Figure 1. We fit the cumulative publication volume of microbial articles and found that it in line with the exponential function, with an *R*^2^ value of 0.9987, indicating an exponentially increasing publication trend.

**Figure 2:**
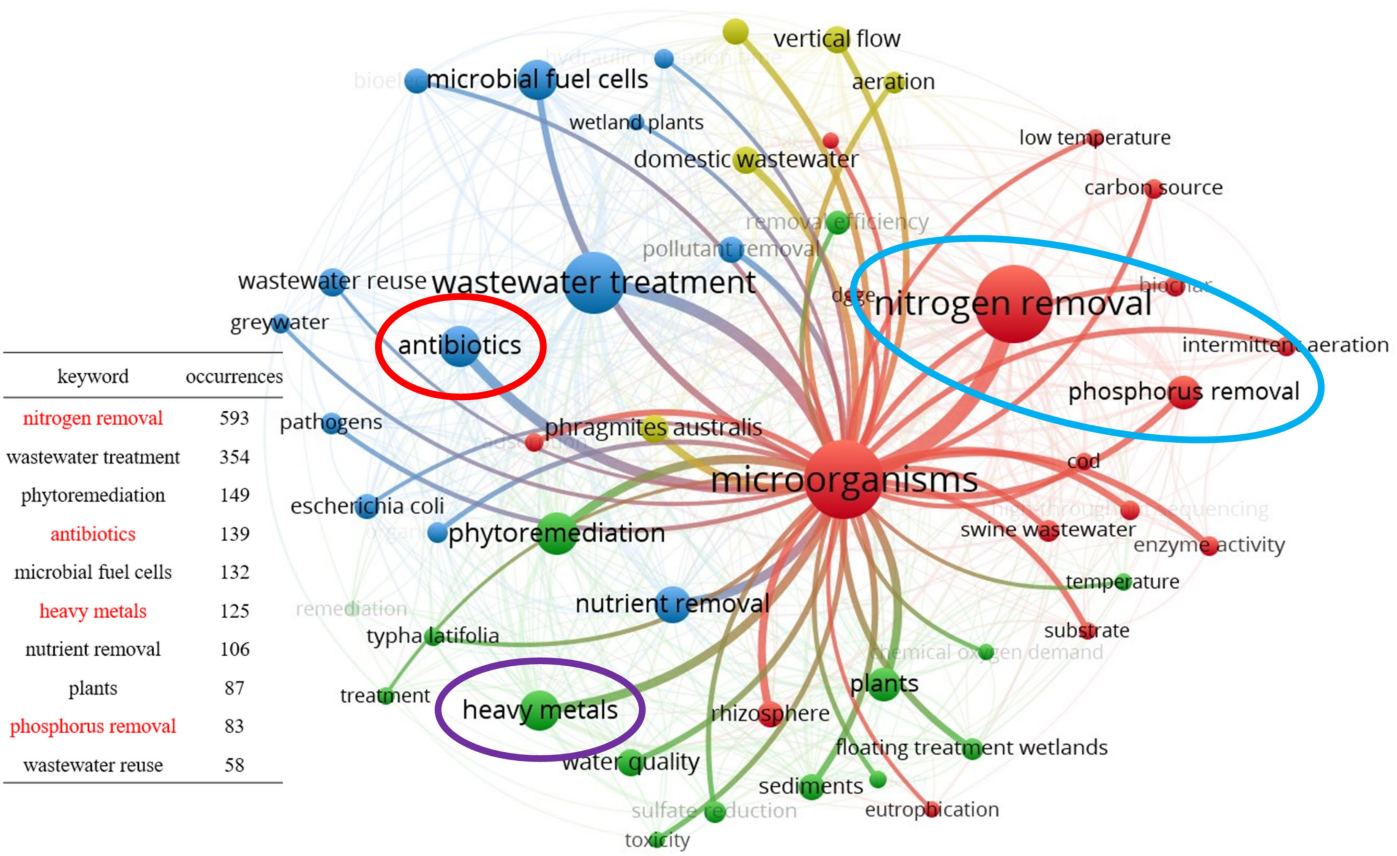
Keyword co-occurrence network visualization map for publications involving microorganisms in CWs.

The second aspect is the analysis of keyword co-occurrence by the VOSviewer software, with the aim to understand the connection among author keywords. After exporting the publications related to microbial from the WOS platform in plain text form, they were analyzed using the keyword co-occurrence function of the VOSviewer software and subsequently merged through the thesaurus file. The final results are shown in Figure 2. This figure shows the top 50 keywords in terms of the number of occurrences number; the high numbers indicate that there are more studies related to them, facilitating the subsequent analysis and summary. Different circles in the figure represent different keywords, and the circle size indicates the number of times the keywords appear, the larger the circle, the more times it appears. The line between the circles indicates that two keywords have appeared together in an article, and the more times they appear together, the thicker the line is. By selecting the keyword “microorganisms,” we could observe the connection between this one and other keywords. Among these keywords, we intercepted the top 10 keywords in terms of number; of these 5 were related to the type of contaminants, namely nitrogen, phosphorus, heavy metals, antibiotics, and nutrients. However, among them, nutrients mainly contain nitrogen and phosphorus (Li et al., 2013), and therefore, this review finally identified nitrogen, phosphorus, heavy metals, and antibiotics as four typical contaminants for a functional microbial summary and diversity analysis.

## Functional Microorganisms

As mentioned above, four pollutants typical for CWs, namely nitrogen, phosphorus, heavy metals, and antibiotics, were identified. Many functional microorganisms play an important role in the treatment of these polluted water. This review provides a summary and functional analysis of these microorganisms. Because the microbial species present in CWs are highly diverse, only the more abundant functional species reported were considered. In addition, only the phylum and genus are summarized and analyzed in this review, as most studies have analyzed microbial species at these two levels.

## Functional Microorganisms in Nitrogen Removal

**Figure 3:**
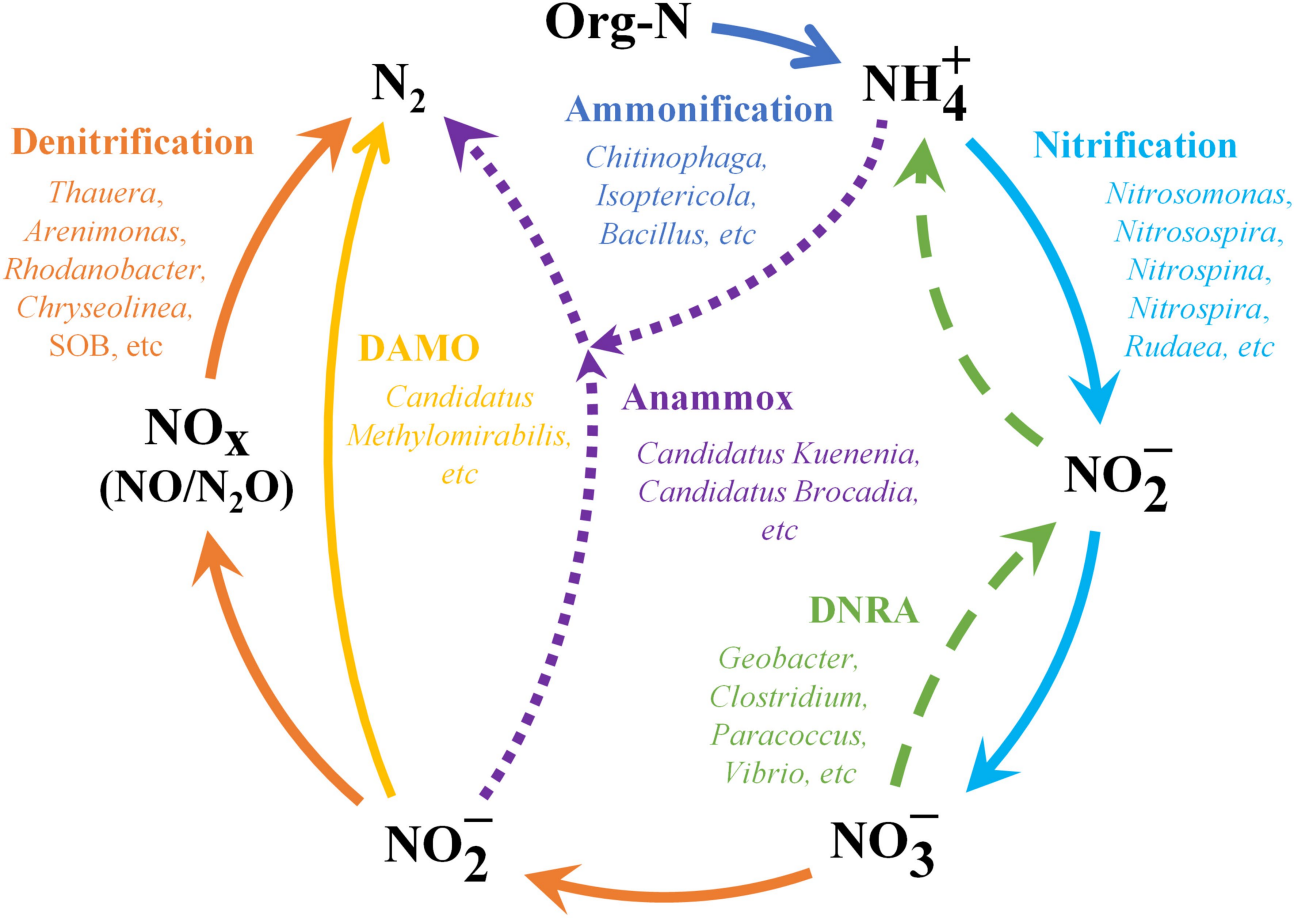
Main processes of nitrogen removal by microorganisms in CWs.

Excess nitrogen discharge into water bodies tends to cause eutrophication and black-odorous, which deteriorates water quality and in turn poses a serious threat to humans and aquatic organisms (Wang et al., 2020d; Zhang et al., 2021c). Biological processes are the key processes in the nitrogen removal mechanisms of CWs, with Tan et al. (2021a) reporting that microorganisms can remove almost 90% of the nitrogen. The main pathways of nitrogen removal by microorganisms in CWs are shown in Figure 3.

Current research generally agrees that nitrogen removal by microorganisms in CWs is mainly accomplished through ammonification, nitrification and denitrification (Hu et al., 2016; Xie et al., 2016; Zhao et al., 2018). Ammonification is the process of converting organic nitrogen (Org-N) in wastewater into NH_4_^+^, which is then removed in other processes (e.g., nitrification, volatilization, and plant uptake; Lee et al., 2014; Xie et al., 2016). As shown in Table 1, the popular genera of ammonifying bacteria include *Chitinophaga*, *Isoptericola*, *Bacillus*, and *Sinorhizobium*. Regarding nitrification and denitrification, the microorganisms use NH_4_^+^ as electron donor during nitrification and oxidize it to NO_2_^−^ and further to NO_3_^−^, which is then used as electron acceptor during denitrification and reduced to N_2_O or N_2_ (Tan et al., 2021b; Zhang et al., 2021c). The microorganisms involved in nitrification can be divided into two categories, ammonia-oxidizing bacteria (AOB) and archaea (AOA) which convert ammonium to nitrite, and nitrite-oxidizing bacteria (NOB) which convert nitrite to nitrate (Zhang et al., 2021b,c). In particular, AOA has a higher adaptability to low ammonia and high salt environments compared to AOB (Wang et al., 2019b; Zhao et al., 2021). This can help the AOA to become the predominant group more quickly and speed up the process of nitrification, as ammonia oxidation is the first and rate-limiting step in nitrification (Wang et al., 2019b). The popular phyla involved in nitrification include *Proteobacteria*, *Nitrospirae*, *Nitrospinae*, and *Thaumarchaeota*. Of these, the *Thaumarchaeota* phylum contains all the currently known AOA (Wang et al., 2019b). Regarding denitrification, *Proteobacteria*, *Bacteroidetes*, *Firmicutes*, and *Actinobacteria* are popular denitrifying bacteria in CWs.

**Table 1:** Functional microorganisms in CWs for nitrogen removal.

Function		Phyla	Genera (Notes)	Nitrogen transformation process	References
Ammonification		*Bacteroidetes, Actinobacteria, Firmicutes, Proteobacteria*	*Chitinophaga, Isoptericola, Bacillus, Sinorhizobium*	Org-N → NH_4_^+^	Xie et al., 2016
Nitrification	Ammonia oxidizing archaea (AOA)	*Thaumarchaeota*	*Nitrososphaera*, *Nitrosopumilus*, *Candidatus Nitrosotalea*, *Candidatus_Nitrosoarchaeum*, *Candidatus_Nitrosopumilus*	NH_4_^+^ → NO_2_^−^	Wang et al., 2019b; Zhao et al., 2021
	Ammonia oxidizing bacteria (AOB)	*Proteobacteria*	*Nitrosomonas*, *Nitrosospira* (Belongs to *Betaproteobacteria*)		Zhao et al., 2016; Huang et al., 2019a
			*Rudaea*, (Belongs to *Gammaproteobacteria*)		
	Nitrite oxidizing bacteria (NOB)	*Proteobacteria*	*Nitrobacter* (Belongs to *Alphaproteobacteria*) *Nitrosococcus* (Belongs to *Gammaproteobacteria*)	NO_2_^−^ → NO_3_^−^	
		*Nitrospirae*	*Nitrospira*		Zhao et al., 2021
		*Nitrospinae*	*Nitrospina*		
		*Chloroflexi*	*Nitrolancea*		Wang et al., 2020d
Denitrification	Traditional denitrification or dissimilatory nitrate to ammonium (DNRA)	*Proteobacteria*	*Bradyrhizobium*, *Hyphomicrobium*, *Rhizobium*, *Rhodobacter*, *Rhodoplanes*, *Paracoccus*, *Methylobacterium*, *Gemmobacter*, *Brevundimonas*, *Roseobacter*, *Azospirillum* (Belongs to *Alphaproteobacteria*)	NO_2_^−^/NO_3_^−^ → N_2_ ↑ (Traditional denitrification) NO_2_^−^/NO_3_^−^ → NH_4_^+^ (DNRA)	Gao et al., 2017; Zhao et al., 2018, 2019, 2020d, 2021; Aguilar et al., 2019; Liu et al., 2020b; Wang et al., 2020d; Jia et al., 2021; Tan et al., 2021b; Zhang et al., 2021d
			*Thauera*, *Comamonas*, *Sulfuritalea*, *Denitratisoma*, *Azoarcus*, *Ralstonia*, *Ferribacterium* (Belongs to *Betaproteobacteria*)		
			*Enterobacter*, *Thermomonas, Arenimonas*, *Rhodanobacter*, *Silanimonas*, *Dokdonella*, *Halomonas*, *Zobellella*, *Thiothrix*, *Vibrio* (Belongs to *Gammaproteobacteria*)		
			*Desulfovibrio*, *Geobacter*, *Sulfuricurvum*		
		*Actinobacteria*	*Propionicella*, *Micropruina*		Zhao et al., 2019
		*Bacteroidetes*	*Maritimimonas*, *Chryseolinea*, *Prolixibacter*, *Paludibacter*, *Terrimonas*		Si et al., 2018; Ajibade et al., 2021; Kowal et al., 2021; Zhao et al., 2021
		*Firmicutes*	*Clostridium*		Zhao et al., 2019
		*Calditrichaeota*	*Calorithrix*		Zhao et al., 2021
	Sulfur autotrophic denitrification (SAD)	*Proteobacteria*	*Thiobacillus, Thiomonas* (Belongs to *Betaproteobacteria*)	S + NO_3_^−^ + NH_4_^+^ → N_2_ ↑ + SO_4_^2−^	Wang et al., 2020a; Zhao et al., 2021
			*Thiohalophilus, Thioalbus* (Belongs to *Gammaproteobacteria*)		
			*Sulfurimonas, Sulfurovum* (Belongs to *Epsilonproteobacteria*)		
		*Bacteroidetes*	*Flavobacteriaceae*		Wang et al., 2020a
	Denitrifying anaerobic methane oxidation (DAMO)	*candidate division NC10*	*Candidatus Methylomirabilis*	CH_4_ + NO_2_^−^ → N_2_ ↑ + CO_2_ ↑	Zhang et al., 2020
Heterotrophic nitrification and aerobic denitrification (HN-AD)		*Proteobacteria*	*Zoogloea, Dechloromonas, Acidovorax, Hydrogenophaga, Ferritrophicum, Propionivibrio* (Belongs to *Betaproteobacteria*)	NH_4_^+^/NO_3_^−^/NO_2_^−^ → N_2_ ↑	Liu et al., 2020b; Tan et al., 2021a; Wang et al., 2022
			*Pseudomonas, Acinetobacter, Aeromonas, Klebsiella* (Belongs to *Gammaproteobacteria*)		
		*Bacteroidetes*	*Flavobacterium, Pedobacter*		Zhao et al., 2016
		*Firmicutes*	*Bacillus*		
Anaerobic ammonia oxidation (anammox)		*Planctomycetes*	*Candidatus_Scalindua, Candidatus Kuenenia, Candidatus Brocadia*	NH_4_^+^ + NO_2_^−^ → N_2_ ↑	Zhao et al., 2021

However, nitrifying bacteria in CWs microbial communities usually face problems of low abundance and weak competitiveness (Tan et al., 2021a). This will result in a longer start-up period required for stable NH_4_^+^ oxidation, making nitrification a limiting step for nitrogen removal (Tan et al., 2021a). In this context, recent studies have highlighted the importance of heterotrophic nitrification and aerobic denitrification (HN-AD) bacteria (Liu et al., 2020b; Tan et al., 2021a; Wang et al., 2022). These bacteria can be responsible for NH_4_^+^ and NO_3_^−^ transformation in the start-up phase of CWs, converting the nitrogen in the aqueous solution to nitrogen gas for complete denitrification (Tan et al., 2021a). Moreover, they grow more rapidly and can dominate quickly (Tan et al., 2021a). The discovery of HN-AD bacteria has changed the traditional theory that nitrification can only be carried out by autotrophic bacteria and denitrification can only take place under anaerobic conditions, which makes it more advantageous in nitrogen removal and organic matter removal (Wang et al., 2022). The HN-AD bacteria reported in the studies mainly included the genera *Dechloromonas*, *Ferribacterium*, *Hydrogenophaga*, *Zoogloea*, and *Aeromonas*. Regarding the denitrification process, new nitrogen removal pathways have also been detected, such as sulfur autotrophic denitrification (SAD) and denitrifying anaerobic methane oxidation (DAMO; Huang et al., 2020b; Wang et al., 2020a). During SAD, sulfur-oxidizing bacteria (SOB) reduce NO_3_^−^ to N_2_, using elemental S, S^2−^, and S_2_O_3_^2−^ as electron donors and NO_3_^−^ as electron acceptors under anaerobic or anoxic conditions (Li et al., 2020a; Wang et al., 2020a). Hence, this pathway may dominate in removing nitrogen of low C/N ratio water due to available electron donors from sulfur and its compounds (Wang et al., 2020a). Most sulfur autotrophic denitrifying bacteria belong to the phylum *Proteobacteria*, with the popular genera being *Thiobacillus* and *Sulfurimonas*. Regarding DAMO, which can reduce NO_2_^−^ to N_2_ under anaerobic conditions using methane (CH_4_) as the electron donor and sole source of carbon (Huang et al., 2020b; Zhang et al., 2020). DAMO can alleviate the greenhouse effect and contribute to reduce the unnecessary byproduct N_2_O in the nitrogen removal process, thus allowing for more environmental benefits (Huang et al., 2020b; Zhang et al., 2020).

In addition to the traditional nitrification–denitrification process of nitrogen removal, there exists a novel pathway—anaerobic ammonia oxidation (anammox; Hu et al., 2016; Kraiema et al., 2019). In this pathway, nitrite is used as an electron acceptor under anaerobic conditions to convert ammonia directly to N_2_ (Zhao et al., 2018; Kraiema et al., 2019). This makes it an alternative denitrification pathway at low oxygen levels and low C/N ratios (Hu et al., 2016). At present, almost all reported anammox bacteria belong to the phylum *Planctomycetes* (Jia et al., 2021; Zhang et al., 2021c). Regarding nitrate, among the different nitrogenous pollutants, nitrate nitrogen is more likely to leach and eventually deteriorate water quality (Li et al., 2021b). Therefore, nitrate removal is important to protect freshwater systems and underground water quality (Li et al., 2021b). In addition to denitrification, there is an alternative pathway for the reduction of nitrate, namely the dissimilatory nitrate reduction to ammonium (DNRA; Rahman et al., 2019). DNRA reduces NO_3_^−^ to available NH_4_^+^ for use by other microorganisms, such as AOB and AOA (Zhang et al., 2021b,c). It has been reported to be more favorable than denitrification under high salinity conditions in sulfide-rich marine and coastal ecosystems (Zhang et al., 2021c). Many studies have found that some denitrifying genera able to execute the DNRA process, such as *Vibrio*, *Clostridium*, and *Desulfovibrio* (Zhang et al., 2021a,c). However, it is still difficult to distinguish denitrifying bacteria from DNRA bacteria, which requires further development of molecular biotechnology. However, currently, denitrifying bacteria and DNRA bacteria are not well distinguished, which requires further development of molecular biotechnology.

Based on the summary in Table 1, the phylum *Proteobacteria* contains a large number of species involved in nitrogen transformation. This phylum is widely distributed in CWs and is the most dominant phylum in most systems, playing an important role in nitrogen removal from different wastewaters (Gao et al., 2017; Si et al., 2018; Zhao et al., 2018). The genera *Nitrosomonas*, *Nitrobacter*, and *Nitrosospira* are associated with nitrification. The genera *Tauera*, *Thiobacillus*, *Thermomonas*, and *Arenimonass* are frequently detected among denitrifying bacteria. The class *Alphaproteobacteria*, *Betaproteobacteria*, and *Gammaproteobacteria* are the dominant class related to nitrogen removal in CWs. They contain large numbers of nitrifying bacteria, AOB, and NOB, which play important ecological functions in CWs and are largely involved in the nitrogen removal (Aguilar et al., 2019; Ajibade et al., 2021).

In addition, there is now a growing number of studies linking functional genes to the functional and quantitative analysis of nitrogen removal microorganisms (Zhao et al., 2020d; Tan et al., 2021b; Zhang et al., 2021b). For example, the abundance of *nrfA*- and *nirK*-carrying microorganisms influenced the denitrification performance of CWs (Zhao et al., 2020d); the abundance of the nitrification functional genes *amoA*-AOA, *amoA*-AOB, and *nxrA* represented the growth status of nitrifying bacteria (Zhao et al., 2021). Currently, the functional gene pools associated with the various processes of nitrogen removal (e.g., nitrification, denitrification, anammox, and DNRA) have been summarized (Tan et al., 2021a,b; Zhang et al., 2021b). Functional genes can essentially analyze the function of microorganisms and provide a feasible approach for us to further study functional microorganisms in CWs.

## Functional Microorganisms in Phosphorus Removal

Phosphorus is one of the main elements causing eutrophication in water bodies (Du et al., 2017; Wang et al., 2021b). Excess phosphorus discharged into the aquatic environment from domestic, agricultural, and industrial sources can also harm aquatic life by altering the pH, lowering oxygen levels, and promoting algal growth (Du et al., 2017; Wang et al., 2021b). Microorganisms play an important role in the removal of phosphorus from CWs and can influence the form of the phosphorus (Wang et al., 2021b). The main microorganisms associated with phosphorus removal in CWs are shown in Table 2.

**Table 2:** Functional microorganisms in CWs for phosphorus removal.

Function	Phyla	Genera (Notes)	Morphology of the removed phosphorus	References
PAO	*Proteobacteria*	*Rhodobacteraceae* (family), *Rhizobiaceae* (family) (Belongs to *Alphaproteobacteria*)	Phosphate	Lv et al., 2021
		*Candidatus Accumulibacter*, *Dechloromonas*, *Rhodocyclus* (Belongs to *Betaproteobacteria*)		Li et al., 2017; Huang et al., 2019a, 2020a; Zheng et al., 2021a
		*Pseudomonas*, *Klebsiella*, *Acinetobacter* (Belongs to *Gammaproteobacteria*)		Du et al., 2017; Tian et al., 2017; Huang et al., 2020a; Zheng et al., 2021a
	*Chloroflexi, Gemmatimonadetes*	*Rhodocyclaceae* (family), *Gemmatimonadacea* (family), *Gemmatimonas*		Wei et al., 2020; Wang et al., 2021b
PSB	*Actinobacteria, Proteobacteria*	*Corynebacterium*, *Enterobacter*	Convert insoluble phosphorus into soluble phosphorus	Wang et al., 2021b
DNPAO	*Proteobacteria*	*Paracoccus* (Belongs to *Alphaproteobacteria*), *Pseudomonadaceae* (family), Pseudomonas, Dechloromonas	Polyphosphate	Huang et al., 2019a; Lv et al., 2021; Wang et al., 2021b
	*Chloroflexi*	*Anaerolineae* (class)		Lv et al., 2021
Solubilize vast tricalcium phosphate through secreting organic acids	*Proteobacteria*	*Delftia* (Belongs to *Betaproteobacteria*)	Phosphate	Li et al., 2020b
Associated with the P element cycle	*Proteobacteria*	*Brevundimonas*, *Pseudorhodoferax*, *Variovorax*, *Panacagrimonas*	Organic phosphoric acid esters/Insoluble phosphate	Wu et al., 2020
	*Chlorobi, Firmicutes, Spirochaetes*	*Chlorobaculum*, *Bacillus*, *Leptospira*		Wu et al., 2020

Biological phosphorus removal in CWs is mainly achieved by phosphorus-accumulating organisms (PAOs), which can absorb phosphate from wastewater and store it in cells under alternating aerobic and anaerobic conditions (Du et al., 2017; Shi et al., 2017; Tian et al., 2017). Under anaerobic conditions, PAOs break down intracellular polyphosphate and take up volatile fatty acids from the environment, which is then stored in the form of polyhydroxyalkanoates (Lv et al., 2021). Under aerobic conditions, PAOs rely on polyhydroxyalkanoates for energy provision and absorb phosphate to form polyphosphate storage (Tian et al., 2017). In general, the amount of phosphorus uptake by PAOs will be greater than the amount of phosphorus released, thus realizing the phosphorus removal process of microorganisms in CWs (Du et al., 2017; Shi et al., 2017; Tian et al., 2017). The main phylum is *Proteobacteria*, which are largely involved in phosphorus removal (Si et al., 2018, 2019; Huang et al., 2020a). Of these, *Alphaproteobacteria*, *Betaproteobacteria*, and *Gammaproteobacteria* contain most of the microbial species associated with biological phosphorus removal (Shi et al., 2017; Huang et al., 2020a; Wei et al., 2020; Lv et al., 2021). The families *Rhodobacteraceae* and *Rhizobiaceae* of the class *Alphaproteobacteria* can absorb volatile fatty acids under aerobic conditions and convert them into poly-β-hydroxyalkanoates, facilitating total phosphorus removal in CWs (Lv et al., 2021). The class *Betaproteobacteria* mainly contains the genera *Candidatus Accumulibacter*, *Dechloromonas*, and *Rhodocyclus*. Of these, the genus *Candidatus Accumulibacter* is considered a typical PAOs and the dominant PAOs in full-scale wastewater treatment plants and laboratory-scale reactors (Huang et al., 2019a). The genus *Dechloromonas* can reduce perchlorate, accumulate polyphosphate, and absorb carbon under anaerobic conditions (Huang et al., 2020a). *Rhodocyclus* have also been shown to have a significant contribution to phosphorus removal (Li et al., 2017). Regarding *Gammaproteobacteria*, three genera, namely *Klebsiella*, *Pseudomonas*, and *Acinetobacter* have been identified in relevant studies (Tian et al., 2017). Of these, *Pseudomonas* has a strong ability to absorb phosphorus from wastewater and store it in its cells as polyphosphate, making it an effective phosphorus-removal microorganism (Huang et al., 2020a). Tian et al. (2017) report that it can remove up to 80.6% of total phosphorus from domestic wastewater. Regarding the genus *Acinetobacter*, it is the first bacteria isolated from biomass with a high phosphorus removal capacity (Du et al., 2017). In addition to *Proteobacteria*, other taxa, such as *Gemmatimonadacea*, that can take up excess phosphate under aerobic conditions (Wang et al., 2021b).

The phosphorus removal efficiency of PAOs mainly depends on the accumulation and consumption of intracellular polyphosphate (Tian et al., 2017), which is directly related to the activities of the enzymes exopolyphosphatase (ppx) and polyphosphate kinase (ppk; Du et al., 2017). The ppx and ppk can catalyze anaerobic phosphorus release and aerobic phosphorus uptake, respectively, to achieve biological phosphorus removal (Tan et al., 2021b). However, high temperatures inhibit their activities; according to a previous study, the optimum temperature ranges from 20.0–35.0°C (Du et al., 2017).

In addition to PAOs, phosphorus-solubilizing bacteria (PSB) and denitrifying phosphorus-accumulating organisms (DNPAO) have also been found in CWs. Example are the genera *Corynebacterium* and *Enterobacter*, which are PSB that secrete organic acids (e.g., oxalic and citric acids) to convert insoluble phosphorus in the soil into soluble phosphorus for plant uptake (Wang et al., 2021b). Regarding DNPAO, it can use NO_3_^−^/NO_2_^−^ as electron acceptors to absorb polyphosphate under anoxic conditions (Wang et al., 2020b). *Alphaproteobacteria* (e.g., the genus *Paracoccus*) and *Anaerolineae* have been reported to be DNPAO (Lv et al., 2021). The genera *Brevundimonas* and *Chlorobaculum* produce organophosphate hydrolases that hydrolyze organophosphate esters, and *Variovorax* can use insoluble phosphate as a phosphorus source for growth (Wu et al., 2020).

## Functional Microorganisms in Heavy Metal Removal

**Figure 4:**
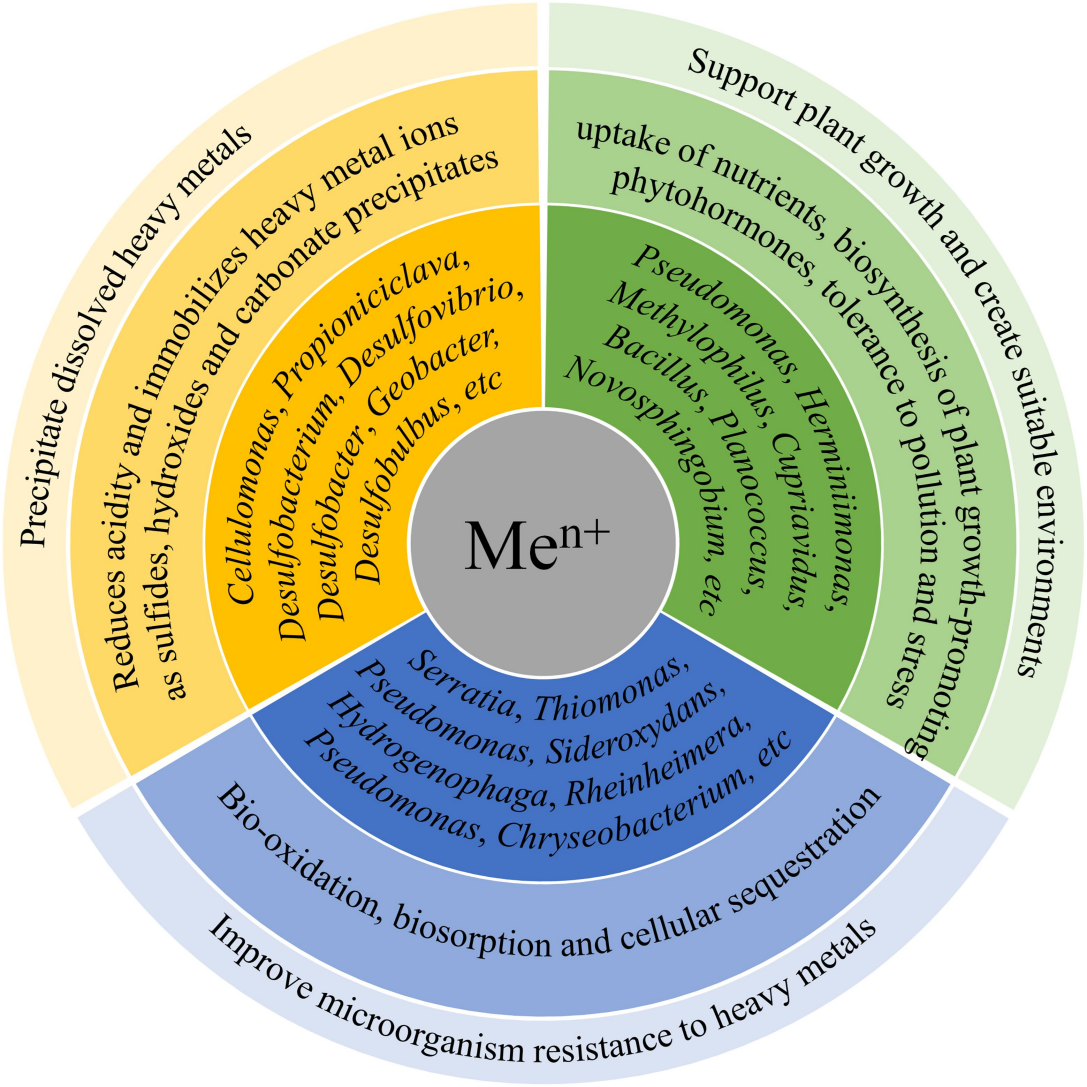
Main mechanisms of heavy metal removal by microorganisms in CWs.

Heavy metals are widely distributed in aquatic systems, difficult to degrade, and can accumulate in the food chain, making them hazardous environmental pollutants (Yu et al., 2020; Chen et al., 2021b). In CWs, microorganisms can be effective in removing heavy metals through mechanisms, such as biosorption, biomineralization, and valence transformation (Si et al., 2019; González Henao and Ghneim-Herrera, 2021). Figure 4 shows the main pathways of heavy metal removal by microorganisms in CWs. The relevant microbial phyla and genera are summarized in Table 3.

**Table 3:** Functional microorganisms in CWs for heavy metal removal.

Pollutant type	Phyla		Genera (Notes)	Removal principle	References
MIW, Especially AMD (Mainly contains Fe^2+^, Cd^2+^, Zn^2+^, Cu^2+^, Cr^2+^ and other heavy metals)	SRB	*Proteobacteria*	*Desulfobacterium*, *Desulforhabdus*, *Desulfobacca*, *Desulforegula*, *Desulfomonile*, *Desulfofustis*, *Desulfovibrio*, *Desulfobacter*, *Desulfobulbus*, *Desulfococcus*, *Desulfocapsa*, *Desulfatirhabdium* (Belongs to *Deltaproteobacteria*)	SO_4_^2−^ + 2CH_2_O → H_2_S + 2HCO_3_^−^ (H_2_S is dissociated into HS^−^ + H^+^) H_2_S + Me^2+^ → MeS ↓ + 2H^+^ (Me^2+^ refers to heavy metal ions)	Chen et al., 2016, 2021c; Sanchez, 2017; Urakawa et al., 2017
		*Firmicutes*	*Desulfotomaculum*, *Desulfosporosinus*		Urakawa et al., 2017; Habe et al., 2020
		*Aquificae*	*Desulfurobacterium*		Urakawa et al., 2017
	Others	*Actinobacteria, Proteobacteria*	*Cellulomonas*, *Propioniciclava*, *Geobacter*		Chen et al., 2021b,c
Fe^2+^	*Proteobacteria*		*Thiomonas*, *Sideroxydans*	Bio-oxidation	Chen et al., 2021c
Cd^2+^, Zn^2+^	*Proteobacteria*		*Serratia*, *Pseudomonas*	Biosorption/Cellular sequestration	Yu et al., 2020
Cu^2+^	*Fusobacteria, Bacteroidetes, Proteobacteria*		*Hydrogenophaga*, *Rheinheimera*	Resistant to heavy metals	Guo et al., 2021
Cd^2+^	*Proteobacteria, Bacteroidetes*		*Pseudomonas*, *Chryseobacterium*		Zhang et al., 2021e
Fe^2+^, Se^4+^	*Firmicutes, Proteobacteria*		*Bacillus*, *Planococcus*, *Pseudomonas*	Support plant growth	Vassallo et al., 2020
Zn^2+^, Ni^2+^, Cd^2+^	*Proteobacteria*		*Herminiimonas*, *Methylophilus*, *Cupriavidus, Novosphingobium*		Syranidou et al., 2018

Among heavy-metal polluted water, mining-impacted water, especially acid mine drainage (AMD), has attracted widespread attention worldwide (Chen et al., 2021b). The AMD generated during and after mining and smelting activities is characterized by high acidity and sulfate and toxic metallic ion enrichment (Chen et al., 2021b,c). Therefore, for AMD remediation, bacterial sulfate reduction in CWs is a key process as it reduces the acidity of AMD and removes heavy metals by immobilizing them as sulfides, hydroxides, and carbonate precipitates (Chen et al., 2021c). The bacteria involved in sulfate reduction are known as sulfate-reducing bacteria (SRB) and they can drive simultaneous sulfate and metal removal as well as acidity neutralization (Habe et al., 2020; Chen et al., 2021b). The majority of SRB belong to the class *Deltaproteobacteria* in the phylum *Proteobacteria*. Among them, the more popular ones include the genera *Desulfovibrio*, *Desulfobacter*, *Desulfobulbus*, and *Desulfurobacterium*. In addition to SRB, other functional microorganisms with complementary ecological niches are also important for the effective remediation of AMD (Chen et al., 2021b,c). For example, members of the genus *Propioniciclava* can use a variety of carbohydrates to produce acetate and propionic acids, driving dissimilatory SBR metabolism (Torregrosa et al., 2019). Regarding the genus *Cellulomonas*, it can protect SRB community from oxygen exposure and also generate low-molecular-weight compounds through saccharification and fermentation to act as electron donors for SRB (Chen et al., 2021b). Therefore, these phyla and genera, which are mainly involved in organic decomposition and sulfate reduction, are the key microbial groups participating in the treatment of AMD in CWs.

Ironically, metal ions generally negatively impact microorganisms by disrupting cell membranes, inhibiting enzyme activity, destroying DNA, and disturbing cellular function (Yu et al., 2020), making tolerance important for the removal of heavy metals by microorganisms. Fe^2+^ can be oxidized to Fe^3+^ by the genera *Thiomonas* and *Sideroxydans*, making it easier to precipitate and thus less hazardous (Chen et al., 2021c). Yu et al. (2020) also found that the genera *Serratia* and *Pseudomonas* screened using Cd^2+^ and Zn^2+^ concentrations gradients showed resistant to these two heavy metals resulting in an increase in removal rates of 10.13 and 8.57%, respectively. The extracellular polymeric substances synthesized by *Pseudomonas* can bind heavy metals and block their diffusion within the biofilm, achieving extracellular sequestration, thereby protecting cells from heavy metal stress (Teitzel and Parsek, 2003; Giovanella et al., 2017). In addition, the cell surface of *Pseudomonas* and *Serratia* could also enhance the adsorption of Cd^2+^ and Zn^2+^ due to the presence of anionic functional groups (Cristani et al., 2012; Limcharoensuk et al., 2015). These findings lead us to infer that the cultivation of resistant microorganisms is a viable approach in heavy metal removal from wastewater and deserves further investigation. However, the way in which *Serratia* reduces the hazard of heavy metals is through secreting several proteins and enzymes such as heavy metal-binding proteins, transporter proteins, amino acids, histidine-binding proteins, and redox enzymes, which can efflux metal ions (Chen et al., 2019). This cannot contribute to heavy metal removal by CWs. Therefore, resistant microorganisms are not exactly the same as functional microorganisms and further research into the mechanisms of heavy metal removal by microorganisms is required to make a determination. Yu et al. (2020) found that functional microorganisms also evolved in the control group that was not inoculated with resistant microorganisms, albeit over a longer period. Most likely, the microbial community structure in the system was spontaneously altered, facilitating resistance to heavy metal stress. In contrast, systems inoculated with resistant microorganisms can experience a less pronounced microbial community evolution to obtain a dominant strain when encountering environments with heavy metals, saving time for biofilm stabilization (Rahman, 2020).

The interactions between microorganisms and plants also greatly affects the removal of heavy metals (González Henao and Ghneim-Herrera, 2021). Microorganisms and plants have long been growing together and microorganisms have more or less established associations with plants (Vassallo et al., 2020). In particular, rhizobacteria and endophytic bacteria can support plant growth through uptake of nutrients (e.g., N, P, Mg, Fe, and Ca), biosynthesis of plant growth-promoting phytohormones, and tolerance to pollution and stress (Syranidou et al., 2018; Vassallo et al., 2020). This can alleviate the toxic stress of heavy metals on plants and can also facilitate the accumulation of heavy metals by plants (Syranidou et al., 2018; Vassallo et al., 2020). In turn, the main function of plants in CWs is to provide additional oxygen and organic matter for microbial growth (Zhou et al., 2013). Thus, good plant growth also provides a more suitable environment for microbial growth (Sturz et al., 2000). This mutualistic interaction facilitates heavy metal removal by CWs. Syranidou et al. (2016) found that inoculation of the *Juncus acutus* with a selected endophytic bacterial consortium removed emergent pollutants and metals faster and more efficiently compared to uninoculated plants. Similarly, Vassallo et al. (2020) isolated eight bacterial strains (belonging to the genera *Bacillus*, *Planococcus*, and *Pseudomonas*) from samples taken from the roots of *Phragmites australis*. They grew well in wastewater with high concentrations of heavy metals (45 mg/l for Fe and 0.09 mg/l for Se), and the higher the concentration of heavy metals, the faster they grow (Vassallo et al., 2020). In conclusion, rhizobacteria and endophytic bacteria have been shown to be reliable functional microorganisms for heavy metal removal as they have sufficient resistance to heavy metals and can enhance phytoremediation efficacy.

## Functional Microorganisms in Antibiotic Removal

Antibiotics are compounds that inhibit the growth of microorganisms (Chen et al., 2020; Xu et al., 2020). They are widely used in human and animal medicine and as animal growth promotors (Chen et al., 2020; Xu et al., 2020). Based on previous studies, antibiotics are now widely present in aquatic environment and that their presence and persistence often cause toxic effects, posing a significant threat to humans, animals, and aquatic habitats (Huang et al., 2017; Shan et al., 2020; Lu et al., 2021). Antibiotic contamination can also lead to the spread of resistance genes, thereby increasing the resistance of microorganisms and reducing the therapeutic potential against human and animal pathogens (Troiano et al., 2018; Chen et al., 2020; Shan et al., 2020). It has been reported that CWs are highly suitable for antibiotic removal, with removal efficiencies as high as 91.8 to 99.5% (Xu et al., 2020). The removal of antibiotics in CWs has undergone a series of complex physical, chemical and biological processes, such as adsorption, precipitation, and microbial degradation (Wang et al., 2019a). Among them, microorganisms are considered to be the driving force for the degradation of antibiotics in CWs (Wang et al., 2019a; Shan et al., 2020; Zheng et al., 2021b). The main functional microorganisms involved in antibiotics removal in CWs are summarized in Table 4.

**Table 4:** Functional microorganisms in CWs for antibiotic removal.

Antibiotic Category		Phyla	Genera (Notes)	Removal principle	References
SAs	SMX	*Proteobacteria*	*Sphingomonas*, *Bradyrhizobium*, *Sphingorhabdus*, *Reyranella*, *Ochrobactrum, Sphingobium*, *Hyphomicrobium* (Belongs to *Alphaproteobacteria*)	Biodegradation or use of antibiotics as carbon source	Syranidou et al., 2017; Man et al., 2020; Sauvetre et al., 2020; Zheng et al., 2021b
			*Acidovorax*, *Ralstonia*, *Azonexus* (Belongs to *Betaproteobacteria*)		Syranidou et al., 2017; Chen et al., 2020
			*Desulfovibrio* (Belongs to *Deltaproteobacteria*)		Ouyang et al., 2021
			*Pseudomonas*, *Luteimonas*, *Enterobacter*, *Acinetobacter* (Belongs to *Gammaproteobacteria*)		Wegrzyn and Felis, 2018; Ouyang et al., 2021; Zheng et al., 2021b
		*Actinobacteria*	*Rhodococcus*, *Microbacterium*, *Arthrobacter*, *Gordonia*, *Nocardioides*, *Streptomyces*		Syranidou et al., 2017; Wegrzyn and Felis, 2018; Sauvetre et al., 2020; Ouyang et al., 2021
		*Firmicutes*	*Bacillus*, *Virgibacillus*		Syranidou et al., 2017; Chen et al., 2020
	SDZ	*Proteobacteria*	*Geobacter* (Belongs to *Deltaproteobacteria*)		Chen et al., 2020
FQ	CIP	*proteobacteria*	*Labrys*, *Bradyrhizobium* (Belongs to *Alphaproteobacteria*)		Syranidou et al., 2017; Santos et al., 2019b; Chen et al., 2020
			*Pseudoxanthomonas* (Belongs to *Gammaproteobacteria*)		Santos et al., 2019b
		*Actinobacteria, Bacteroidetes*	*Nocardioides*, *Dysgonomonas*		Alexandrino et al., 2017; Santos et al., 2019b
	OFL	*Proteobacteria*	*Rhizobacter*, *Uliginosibacterium* (Belongs to *Betaproteobacteria*)		Lu et al., 2021
		*Actinobacteria, Bacteroidetes*	*Arthrobacter*, *Bacteroides*		Tong et al., 2020; Lu et al., 2021
	ENR	*Bacteroidetes*	*Alkaliflexus*, *Dysgonomonas*		Alexandrino et al., 2017; Santos et al., 2019b
DCF		*Proteobacteria*	*Labrys*, *Sphingobium* (Belongs to *Alphaproteobacteria*)		Wegrzyn and Felis, 2018; Sauvetre et al., 2020
		*Actinobacteria*	*Microbacterium*, *Brevibacterium*, *Streptomyces*		Wegrzyn and Felis, 2018; Sauvetre et al., 2020
		*Fungi*	*Trametes*		Wegrzyn and Felis, 2018
Ampicillin		*Proteobacteria*	*Luteimonas*, *Pseudoxanthomonas* (Belongs to *Gammaproteobacteria*)		Santos et al., 2019b; Zheng et al., 2021b
Tetracycline		*Proteobacteria*	*Novosphingobium*		Santos et al., 2019b; Lu et al., 2021
TCS		*Proteobacteria*	*Pseudomonas*, *Alcaligenes*, *Stenotrophomonas*, *Methylococcales* (order) (Belongs to *Gammaproteobacteria*)		Liu et al., 2016
CEF		*Bacteroidetes*	*Dysgonomonas*		Alexandrino et al., 2017

Sulfonamides (SAs), including sulfamethoxazole (SMX) and sulfadiazine (SDZ), are widely used in animal agriculture and human health care and are the most common residual antibiotics in almost all environmental compartments (Ouyang et al., 2021). They can significantly inhibit bacterial populations, such as *Desulfarculus*, denitrifying bacteria, and *Syntrophobacter*, affecting the sulfur and nitrogen cycles (Man et al., 2020). Microbial-mediated degradation can significantly contribute to the removal of SAs in CWs, both under aerobic and anaerobic conditions (Chen et al., 2020). For example, under aerobic conditions, *Bacillus* and *Geobacter* can degrade SAs (Chen et al., 2020). The genus *Bacillus*, belonging to the phylum *Firmicutes*, can be enriched under SAs stress, degrading SMX to NH_4_^+^ and further to NO_3_^−^ (Liu et al., 2018). *Geobacter*, a member of the phylum *Proteobacteria*, is considered a potential SDZ degrader (Zhang et al., 2019a). The genus *Microbacterium* in the phylum *Actinobacteria* can also use SMX as the sole carbon source under aerobic conditions (Sauvetre et al., 2020; Ouyang et al., 2021). The molecular mechanism of SMX catabolism by *Microbacterium* is initiated by ipso-hydroxylation, followed by NADH-dependent hydroxylation of the carbon atom attached to the sulfonyl group, which leads to the release of sulfite, 3-amino-5-methylisoxazole, and benzoquinone imine, of which the latter is converted to 4-aminophenol (Ricken et al., 2013). As for the genus *Bradyrhizobium* can accelerate SAs removal under anaerobic conditions (Chen et al., 2020). In CWs, the three main phyla involved in the degradation of SAs are *Proteobacteria*, *Actinobacteria* and *Firmicutes*, and some key genera, such as *Pseudomonas*, may metabolize glucose and subsequently attenuate SMX by co-metabolism of organic matter and SMX (Zheng et al., 2021b). In addition, *Desulfovibrio* also plays a key role in SMX transformation and can transform SMX alone (Ouyang et al., 2021).

In addition to SAs, common antibiotics include fluoroquinolones (FQ) and cephalosporin (CP), two of which are the most widely used antimicrobials drugs worldwide (Alexandrino et al., 2017). Among them, FQ include ciprofloxacin (CIP), ofloxacin (OFL), and enrofloxacin (ENR); CP include ceftiofur (CEF). For these types of antibiotics, Amorim et al. (2014) investigated the soil bacterium *Labrys portucalensis F11* in minimal medium supplemented with acetate as an additional carbon source and demonstrated its ability to degrade a range of FQ (e.g., CIP). Similarly, Lin et al. (2016) suggested that the genus *Arthrobacter* can dissipate FQ (e.g., OFL) as an additional carbon and energy source. The genus *Dysgonomonas* has also been shown to biodegrade ENR and CEF (Alexandrino et al., 2017).

In addition, the first Watch List of the EU Water Framework Directive [European Commission (EC)2015] identifies the anti-inflammatory diclofenac (DCF) and the antibiotic SMX as two emerging contaminants (Sauvetre et al., 2020). For DCF, Bessa et al. (2017) demonstrated that strain *Brevibacterium* sp. *D4* could biodegrade 35% of 10 mg/l of DCF as the sole carbon source. Moreira et al. (2018) also reported that the bacterial strain *Labrys. portucalensis F11* degraded 70% of 34 μM of DCF, supplied as the sole carbon source, after 30 days of cultivation. Regarding the microorganisms associated with the degradation of other antibiotics, such as ampicillin, tetracycline, triclosan (TCS), and ceftiofur (CEF) are listed in Table 4.

As shown in Table 4, the vast majority of functional microorganisms related to antibiotic removal belong to the *Proteobacteria*, *Acidobacteria*, and *Bacteroidetes* phyla, probably due to the presence of degradation genes (Liu et al., 2019). According to Huang et al. (2017), the phylum most significantly related to antibiotic removal is *Proteobacteria* phylum, followed by *Bacteroidetes* and *Actinobacteria*. Of these, *Betaproteobacteria* of the phylum *Proteobacteria* have been shown to be effective in addressing the global antibiotic resistance issue (Alexandrino et al., 2017; Shan et al., 2020).

However, functional microorganisms may develop antibiotic resistance in the process of degrading antibiotics and may even cover functions other than antibiotic resistance (Alonso et al., 2001; Troiano et al., 2018). Antibiotic resistance may be inherent to microorganisms or may arise through horizontal gene transfer from donor bacteria, phages, or free DNA (Alonso et al., 2001; Troiano et al., 2018; Santos et al., 2019a). An increase in antibiotic resistant microorganisms may lead to a decrease in the therapeutic potential of antibiotics, thus making it more difficult to treat microorganisms’ infections (Santos et al., 2019a). Notably, an induction of antibiotic resistance genes has been reported with the effective removal of antibiotics by CWs (Liu et al., 2019). Therefore, it is a great challenge for CWs to avoid the induction of antibiotic resistance genes while effectively removing antibiotics.

## Emerging Pollutants

In addition to the above four typical pollutants, CWs are also used to remove some emerging pollutants, such as hormones, pesticides, food additives, flame retardants, nanoparticles, and persistent organic pollutants (e.g., polychlorobiphenyls and polycyclic aromatic hydrocarbons; Rajan et al., 2019; Vymazal et al., 2021; Yin et al., 2021). Biodegradation is generally considered as one of the important processes responsible for these emerging pollutants removal (Vymazal et al., 2021). Zhang et al. (2021e) found that *Firmicutes*, *Clostridia*, and *Acetobacterium* were able to tolerate abiotic stresses and thus degrade chlorpyrifos into carbon sources. Liu et al. (2020b) demonstrated that *Pseudomonas*, *Duganella*, and *Sphingobium* are resistant to the threat of organophosphate flame retardants [tris (2-chloroethyl) phosphate, tris (1-chloro-2-propyl) phosphate, and tricresyl phosphate] and have the ability to biodegrade. Ahmad et al. (2019) also showed that various genera, such as *Flavobacteriaceae*, *Novosphingobium*, and *Mycobacterium* can degrade polycyclic aromatic hydrocarbons in a diverse environment. However, there is still relatively little research on these emerging pollutants removed by microorganisms in CWs, so this section is not the focus of this review and more research is needed in the future to focus on the degradation and removal mechanisms of these emerging pollutants by microorganisms.

## Microbial Alpha Diversity Analysis

In addition to the composition and structure of microorganisms, the diversity of microbial communities can also influence the performance of CWs in removing pollutants (Zhang et al., 2019b). Therefore, this review explored the effect of four typical pollutants on the microbial diversity of CWs by counting the values of microbial diversity in different studies. As alpha diversity can reflect the species diversity of microbial communities within a given region, this review has chosen to represent the diversity of microbial communities through the value of alpha diversity (Pitacco et al., 2019; Laliberte et al., 2020).

**Figure 5:**
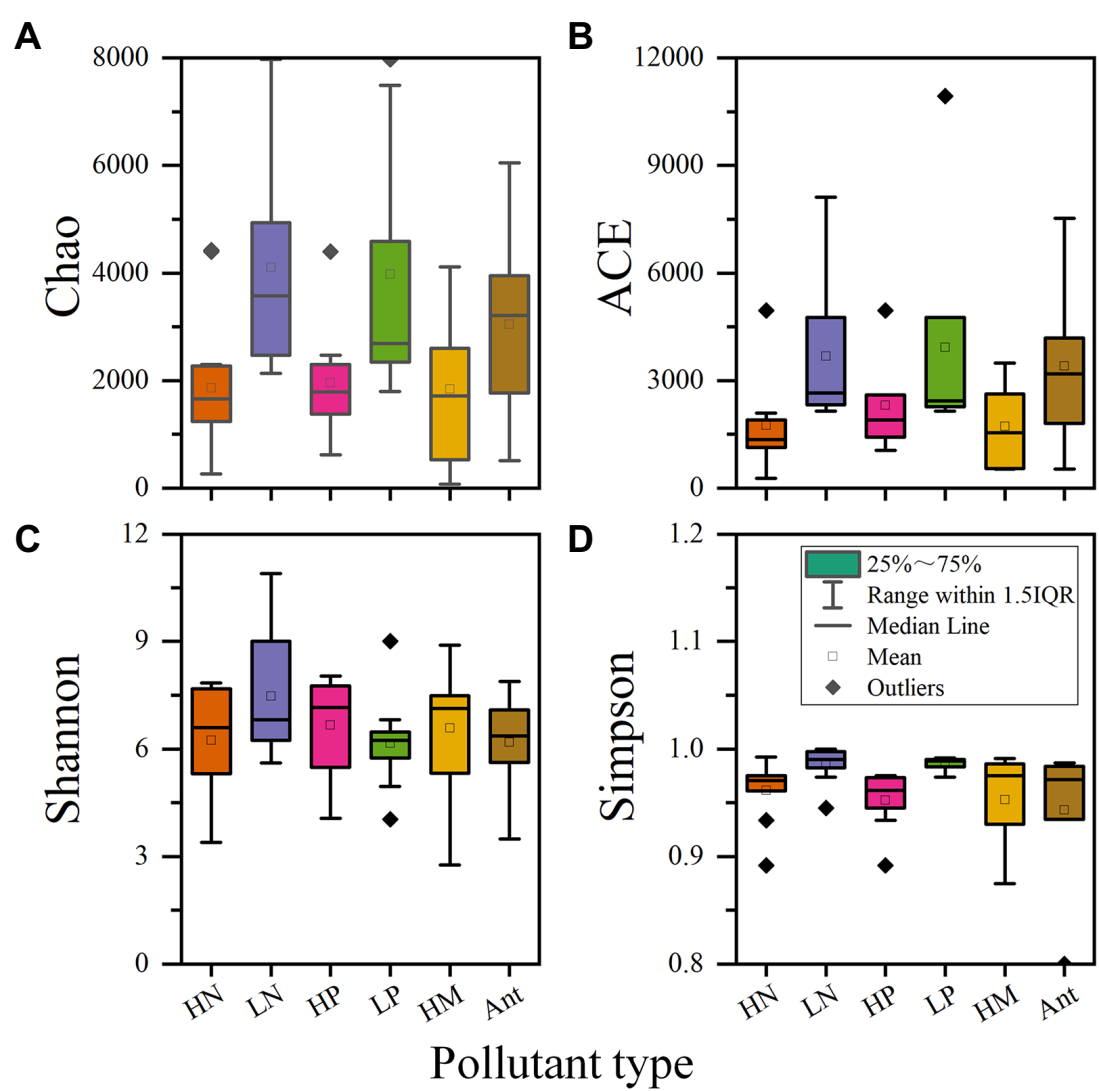
Effects of four typical pollutants on microbial alpha diversity in CWs. **(A)** Chao index; **(B)** ACE index; **(C)** Shannon index; **(D)** Simpson index (HN: high concentration of nitrogen; LN: low concentration of nitrogen; HP: high concentration of phosphorus; LP: low concentration of phosphorus; HM: heavy metal; Ant: antibiotic).

To better reflect the influence of pollutant concentration on microbial diversity, the pollutants nitrogen and phosphorus were classified into high and low concentrations. Since CWs are generally used for deep treatment of the tailwater of wastewater treatment plants, wastewater with total nitrogen concentrations exceeding 20 mg/l and total phosphorus concentrations exceeding 1.5 mg/l was designated as high concentrations with reference to the Discharge Standard of Pollutants for Municipal Wastewater Treatment Plant (GB 18918–2002) - level B standard. Table 5 shows the specific values of microbial alpha diversity in CWs considering the four typical pollutants (nitrogen, phosphorus, heavy metals, and antibiotics). To better visualize and compare, box plots (Figure 5) were generated using the median value of alpha diversity in Table 5 as the data.

**Table 5:** Alpha diversity of microorganisms in CWs treated different pollutant.

Alpha Diversity	Richness		Diversity		Concentration of pollutants (mg/l)	References
Pollutant type	Chao1	ACE	Shannon	Simpson		
High concentration of nitrogen	266.500 ± 62.500	269.000 ± 59.000	3.39500 ± 0.58500		NH_4_^+^ = 35.000, TN = 40.000	Zhao et al., 2016
	621.250 ± 115.150		4.07000 ± 0.71000		NO_3_^−^ = 4.395 ± 0.695, NH_4_^+^ = 79.945 ± 1.805, TN = 87.100 ± 2.620	Li et al., 2019
	841.500 ± 163.500	1048.950 ± 190.230	6.72500 ± 0.17500	0.96814 ± 0.00316	NH_4_^+^ = 89.200	Xu et al., 2021
	1237.117 ± 230.722	1224.746 ± 245.1905	6.66750 ± 1.25450	0.96100 ± 0.02800	NH_4_^+^ = 18.000, NO_3_^−^ = 6.000, TN = 24.000	Xia et al., 2020
	1296.915 ± 59.345	1292.191 ± 62.723	5.27000 ± 0.17400	0.98615 ± 0.00345	NO_3_^−^ = 50.000	Zhao et al., 2019
	1375.965 ± 257.945	1422.860 ± 244.730	5.78500 ± 0.78500		NH_4_^+^ = 226.284	Lv et al., 2021
	1586.500 ± 214.500		7.67250 ± 0.13250		NH_4_^+^ = 20.140 ± 0.420, NO_3_^−^ = 39.290 ± 0.730	Deng et al., 2019
	1725.500 ± 108.500	1725.500 ± 108.500	7.84500 ± 0.09500	0.97331 ± 0.00690	NH_4_^+^ = 115.000, NO_3_^−^ = 182.000	Zhao et al., 2020d
	1755.075 ± 92.235		7.68319 ± 0.43451	0.97313 ± 0.00814	NH_4_^+^ = 26.250 ± 11.250, NO_3_^−^ = 10.500 ± 4.500, TN = 43.750 ± 18.750	Xiao et al., 2020
	2001.110 ± 883.090	2091.605 ± 913.475	7.15500 ± 2.15500	0.89171 ± 0.10240	NH_4_^+^ = 112.580	Wang et al., 2021b
	2274.150 ± 56.660		7.76500 ± 0.20500	0.97500 ± 0.00500	NH_4_^+^ = 15.170 ± 0.804, TN = 21.910 ± 1.190	Huang et al., 2018
	2299.615 ± 366.595		5.31000 ± 0.42100	0.96165 ± 0.02155	NH_4_^+^ = 20.000, NO_3_^−^ = 1.200, TN = 35.000	Yang et al., 2021
	4398.800 ± 804.200	4960.050 ± 535.150	5.48500 ± 1.35500	0.93350 ± 0.06250	NH_4_^+^ = 29.900, TN = 39.000	Zheng et al., 2020
	4422.000 ± 315.000		6.51850 ± 0.25450	0.99235 ± 0.00295	NH_4_^+^ = 18.680, TN = 60.360	Pelissari et al., 2018
Low concentration of nitrogen	2133.745 ± 127.930	2144.504 ± 139.730	5.84550 ± 0.15450	0.99150 ± 0.00250	NH_4_^+^ = 5.315 ± 2.345, TN = 13.425 ± 5.785	Li et al., 2021a
	2346.600 ± 216.230	2330.600 ± 228.940	6.28000 ± 0.23000		NH_4_^+^ = 1.290 ± 0.020, NO_3_^−^ = 7.380 ± 0.130, NO_2_^−^ = 0.110 ± 0.010, TN = 14.680 ± 0.250	Jia et al., 2021
	2468.095 ± 837.095	2602.635 ± 971.635	8.0350 ± 1.0650	0.94488 ± 0.03924	TN = 20.000	Huang et al., 2019a
	2686.500 ± 317.500	2655.500 ± 303.500	6.24500 ± 0.74500	0.97360 ± 0.02460	NH_4_^+^ = 0.170, TN = 2.480	Wang et al., 2020c
	3119.050 ± 215.950	3110.500 ± 252.140	5.60500 ± 0.27500	0.98225 ± 0.00595	NO_3_^−^ = 12.000, NH_3_^+^ = 8.000	Ajibade et al., 2021
	3574.000 ± 75.000		10.89500 ± 0.03500	0.99874 ± 0.00002	NH_4_^+^ = 4.000, NO_3_^−^ = 10.000	Qin et al., 2021
	4592.500 ± 269.500	4765.500 ± 259.500	6.47000 ± 0.22000	0.98900 ± 0.00100	NH_4_^+^ = 1.630 ± 0.090, NO_3_^−^ = 10.410 ± 1.660, TN = 12.680 ± 1.320	Tong et al., 2019
	4932.250 ± 175.350		9.80000 ± 0.33000	1.00000 ± 0.00000	NH_4_^+^ = 9.110, NO_3_^−^ = 9.530, TN = 19.050	Wei et al., 2020
	6924.040 ± 1255.720	8110.050 ± 935.750	7.16000 ± 0.20000	0.99730 ± 0.00170	NH_4_^+^ = 0.960	Zhao et al., 2020c
	4393.430		9.00625	0.99018	NH_4_^+^ = 1.500, NO_3_^−^ = 10.500	Zhang et al., 2021e
	7972.000 ± 186.000		6.81750 ± 0.35750	0.92500 ± 0.01500	NH_4_^+^ = 2.408 ± 2.350, NO_3_^−^ = 1.885 ± 0.925, NO_2_^−^ = 0.105 ± 0.091	Ma et al., 2018
High concentration of phosphorus	621.250 ± 115.150		4.07000 ± 0.71000		TP = 10.525 ± 0.715	Li et al., 2019
	841.500 ± 163.500	1048.950 ± 190.230	6.72500 ± 0.17500	0.96814 ± 0.00316	PO_4_^3−^ = 44.000	Xu et al., 2021
	1375.965 ± 257.945	1422.860 ± 244.730	5.78500 ± 0.78500		PO_4_^3−^ = 19.554	Lv et al., 2021
	1725.500 ± 108.500	1725.500 ± 108.500	7.84500 ± 0.09500	0.97331 ± 0.00690	PO_4_^3−^ = 4.387	Zhao et al., 2020d
	1755.075 ± 92.235		7.68319 ± 0.43451	0.97313 ± 0.00813	TP = 5.250 ± 2.250	Xiao et al., 2020
	1788.145 ± 157.145		7.47000 ± 0.50000	0.95000 ± 0.04000	TP = 3.000	Huang et al., 2020a
	2001.110 ± 883.090	2091.605 ± 913.475	7.15500 ± 2.15500	0.89171 ± 0.10240	PO_4_^3−^ = 17.500	Wang et al., 2021b
	2274.150 ± 56.660		7.76500 ± 0.20500	0.97500 ± 0.00500	TP = 2.810 ± 0.170	Huang et al., 2018
	2299.615 ± 366.595		5.31000 ± 0.42100	0.96165 ± 0.02155	TP = 5.000	Yang et al., 2021
	2468.095 ± 837.095	2602.635 ± 971.635	8.03500 ± 1.06500	0.94488 ± 0.03924	TP = 3.000	Huang et al., 2019a
	4398.800 ± 804.200	4960.050 ± 535.150	5.48500 ± 1.35500	0.93350 ± 0.06250	TP = 3.600 ± 0.900	Zheng et al., 2020
Low concentration of phosphorus	4592.500 ± 269.500	4765.500 ± 259.500	6.47000 ± 0.22000	0.98900 ± 0.00100	PO_4_^3−^ = 0.800 ± 0.070	Tong et al., 2019
	2346.600 ± 216.230	2330.600 ± 228.940	6.28000 ± 0.23000		TP = 0.210 ± 0.010	Jia et al., 2021
	2133.745 ± 127.930	2144.504 ± 139.730	5.84550 ± 0.15450	0.99150 ± 0.00250	TP = 0.505 ± 0.325	Li et al., 2021a
	2686.500 ± 317.500	2655.500 ± 303.500	6.24500 ± 0.74500	0.97360 ± 0.02460	TP = 0.260	Wang et al., 2020c
	7492.815 ± 1030.225	10929.450 ± 1071.540	5.74500 ± 0.46500	0.98350 ± 0.00890	TP = 0.550 ± 0.300	Kang et al., 2017
	2355.050 ± 745.750	2442.000 ± 656.000	4.95600 ± 0.93300		TP = 1.200 ± 0.300	Wang et al., 2020a
	1796.650 ± 461.450	2269.500 ± 380.500	4.03650 ± 0.52850			
	4393.430		9.00625	0.99018	TP = 1.500	Zhang et al., 2021e
	7972.000 ± 186.000		6.81750 ± 0.35750	0.92500 ± 0.01500	PO_4_^3−^ = 0.407 ± 0.025	Ma et al., 2018
Heavy Metal	961.900	969.000	7.08000	0.97900	Control group (NH_4_^+^ = 114.600, PO_4_^3−^ = 17.900)	Liu et al., 2020a
	510.000 ± 109.600	527.600 ± 108.600	4.79950 ± 1.34850	0.87500 ± 0.09600	Ni = 2.000, 5.000, 10.000, 30.000	
	5296.025 ± 164.585		9.86543 ± 0.10423	0.99410 ± 0.00457	Control group (NH_4_^+^ = 141.580, NH_2_^−^ = 17.140, NO_3_^−^ = 43.330, PO_4_^3−^ = 13.170)	Xiao et al., 2021
	2443.830 ± 238.130		8.89639 ± 0.67463	0.99107 ± 0.00491	Ni = 0.100, 1.000	
	516.425 ± 201.145	509.965 ± 192.635	5.14000 ± 0.43000	0.90500 ± 0.01500	Control group (NO_3_^−^ = 50.500, NH_4_^+^ = 75.428, PO_4_^3−^ = 6.581)	Wang et al., 2020b
	532.555 ± 132.355	545.470 ± 135.490	5.32000 ± 0.25000	0.93000 ± 0.02000	Zn = 24.769	
	3186.590 ± 456.720	3177.470 ± 474.670	6.56500 ± 0.36500	0.99425 ± 0.00325	Control group (NO_3_^−^ = 4.195, NH_4_^+^ = 2.904, PO_4_^3−^ = 1.727)	Wang et al., 2021a
	2602.910 ± 851.850	2621.995 ± 887.785	6.03500 ± 0.36500	0.98605 ± 0.00755	Cr = 0.100 mmol/l	
	1288.690 ± 581.040	1353.650 ± 622.490	7.69500 ± 1.47500	0.96500 ± 0.02500	Control group (NO_3_^−^ = 50.500, NH_4_^+^ = 75.428, PO_4_^3−^ = 6.581)	Zhao et al., 2020a
	1353.210 ± 806.080	1327.970 ± 478.900	7.12500 ± 1.77500	0.93000 ± 0.06000	Pb = 5.000	
	73.350 ± 42.150		2.76750 ± 0.72550	0.88450 ± 0.09650	As = 20.000, Zn = 15.000	Arroyo et al., 2013
	1962.290 ± 48.290	2032.945 ± 118.605	7.48500 ± 0.75500	0.96200 ± 0.01600	Cu = 2.000 ± 0.170, Zn = 4.000 ± 0.210, Cd = 0.100 ± 0.010, Co = 2.000 ± 0.230, Ni = 0.500 ± 0.040, Pb = 0.500 ± 0.270	Si et al., 2019
	3387.500 ± 461.500	3491.000 ± 453.000	7.12000 ± 0.82000	0.97500 ± 0.01500	Cu = 4.880 ± 0.080, Zn = 5.060 ± 0.210, Cd = 5.170 ± 0.170, Cr = 5.650 ± 0.580	Chen et al., 2021c
	1469.050 ± 182.450	1540.050 ± 182.300	7.44500 ± 0.39500	0.98500 ± 0.00500	Cr = 0.500, 1.000, 2.000, 4.000, 8.000, 16.000	Zhang et al., 2019b
	4217.190		8.77863	0.98390	Cd = 200.000 μg/l	Zhang et al., 2021e
	4019.520		8.88151	0.98791	Cd = 200.000 μg/l, chlorpyrifos =200.000 μg/l	
Antibiotic	4592.500 ± 269.500	4765.500 ± 259.500	6.47000 ± 0.22000	0.98900 ± 0.00100	Control group (NO_3_^−^ = 10.410 ± 1.660, NH_4_^+^ = 1.630 ± 0.090, TN = 12.680 ± 1.320, PO_4_^3−^ = 0.800 ± 0.070)	Tong et al., 2019	
	4088.500 ± 702.500	4184.000 ± 755.000	6.35000 ± 0.26000	0.98700 ± 0.00400	Ofloxacin = 0.100, 10.000, 1000.000 μg/l		
	1415.915 ± 733.675	1471.010 ± 759.730	4.61000 ± 0.73000	0.95220 ± 0.03370	Control group (NO_3_^−^ = 10.590, NH_4_^+^ = 20.630, TN = 32.340, PO_4_^3−^ = 4.310)	Zheng et al., 2021b	
	1794.070 ± 641.170	1805.390 ± 620.340	5.17000 ± 0.51000	0.98010 ± 0.00720	Sulfamethoxazole =100.000 μg/l		
	485.356 ± 186.811	497.745 ± 173.830	3.72600 ± 0.64200	0.93750 ± 0.02150	Control group (NO_3_^−^ = 50.000, NH_4_^+^ = 76.420, TN = 12.680 ± 1.320, PO_4_^3−^ = 6.600)	Lu et al., 2021	
	509.085 ± 253.085	528.041 ± 240.405	3.48900 ± 1.31900	0.80000 ± 0.18100	Levofloxacin = 0.100, 0.200, 0.300, 0.500, 1.000, 10.000, 100.000 μg/l			
	3425.000 ± 275.000	3420.000 ± 220.000	7.63000 ± 0.16000	0.93450 ± 0.00850	Ciprofloxacin = 99.400 ± 8.300 μg/l, Azithromycin = 1313.900 ± 63.600 μg/l, Oxytetracycline =972.350 ± 39.950 μg/l	Wang et al., 2019a	
	6048.560 ± 1435.820	7528.120 ± 1563.730	6.06500 ± 0.53500	0.98371 ± 0.00952	Triclosan = 60.000 μg/l	Liu et al., 2016	
	3804.525 ± 72.185		7.87500 ± 0.53500	0.97140 ± 0.01420	ciprofloxacin hydrochloride = 50.000 μg/l, Sulfamethoxazole = 50.000 μg/l	Yuan et al., 2020	
	1748.150 ± 55.850		6.53450 ± 0.73150	0.94500 ± 0.02800	Sulfadiazine = 4.000	Song et al., 2018	
	2987.970 ± 261.210	2965.010 ± 250.950	6.38100 ± 0.30700		Enrofloxacin = 46.550 ± 20.850 ng/l, Sulfamethoxazole =1 37.600 ± 73.600 ng/l	Huang et al., 2019b

*Alpha diversity is mainly related to two factors. One is richness*, i.e.*, the number of species; and the other is evenness*, i.e.*, the relative abundance of different species. The Chao1 and ACE indices reflect richness; and the Shannon and Simpson indices are a combination of richness and evenness that reflects diversity. Higher values of these indices represent a higher richness or diversity of microbial communities*.

## Effects of Nitrogen and Phosphorus on Alpha Diversity

As shown in Figure 5, nitrogen and phosphorus have similar effects on alpha diversity, so we put them together for analysis. Regarding richness, at high concentrations of nitrogen and phosphorus, both the Chao1 index and the ACE index were significantly lower than at low concentrations, suggesting that microbial richness is severely reduced in environments with high concentrations of these pollutants. Most likely, microorganisms not involved in pollutant removal are eliminated or suppressed in these extreme environments, resulting in fewer microbial species (Xiao et al., 2020). The closer data for high concentrations of nitrogen and phosphorus also indicate that certain specific microbial species may have formed, resulting in similar richness levels in different studies. On the contrary, at low concentrations of nitrogen and phosphorus, although the values of both Chao1 and ACE indexes were higher, the data were more scattered. This may be due to the dominance of other influencing factors, such as C/N ratio (Jia et al., 2021) and substrate type (Ajibade et al., 2021).

Regarding diversity, it is evident from the Simpson index that high concentrations of nitrogen lead to a decrease in diversity, i.e., to the emergence of dominant populations. This corresponds with the richness analysis. Apparently, high nitrogen concentrations facilitate the growth of microorganisms associated with nitrogen cycling and suppress the growth of microorganisms of other functions (Xiao et al., 2020). Interestingly, the Shannon index was high but the Simpson index was low for high phosphorus concentrations, with the opposite pattern for low phosphorous levels. This may be explained by the high sensitivity of the Shannon index to community richness (Zhang et al., 2019b). Low phosphorous concentrations allow for a consistently high level of species diversity, reflecting no large differences in abundance among microorganisms with different functions in the absence of significantly prominent contaminants.

The combined richness and diversity indices show that high concentrations of nitrogen and phosphorus lead to varying reductions in both indices, reflecting the presence of significantly dominant populations. These populations are the functional microorganisms that play an important role in the removal of nitrogen and phosphorus.

## Effects of Heavy Metals on Alpha Diversity

Heavy metals can significantly decrease microbial richness. This can be explained by the toxicity of heavy metals to microorganisms and the inability of microorganisms to directly degrade heavy metals (Bianchi et al., 2020). In this sense, the presence of heavy metals had a selection effect on microorganisms in CWs, and microbial succession occurred in CWs over time, where the enrichment and structural optimization of dominant species may lead to the reduction in richness and diversity (Xiao et al., 2021). However, in contrast to the case of high concentrations of nitrogen and phosphorus, the difference between the data for heavy metals is larger, resulting in a larger box in the box plot. The most likely reasons for this are that the toxicity of different types of heavy metals to microorganisms may vary and that different microorganisms have different resistance levels to heavy metals.

Regarding the effect on diversity, although the median of heavy metals can be high, the data are scattered, with a relatively large gap between the maximum and the minimum values. The occurrence of low values is easy to understand because heavy metals screen and selectively enrich microbial communities (Jia et al., 2021; Xiao et al., 2021), and most studies on heavy metals select microorganisms isolated from plant roots or screened from wastewater with high concentrations of heavy metals as functional microorganisms, resulting in the formation of dominant populations and in a lower diversity index. The high values can be explained by the microbial community being under pressure from heavy metals and the species within the community all evolving towards high heavy metal resistance (Yu et al., 2020). As a result, the abundance of different species is gradually increasing and the community as a whole is more even, so that there are no clearly dominant populations. In addition, during the treatment of heavy metals, key microorganisms require a variety of other microorganisms to cooperate and complement them (Chen et al., 2021b), which can also lead to a higher diversity. For example, the section “Functional Microorganisms in Heavy Metal Removal” mentioned a variety of microorganisms that can enhance the metabolism of SRB or provide them with electron donors, thus enhancing the bacterial sulfate reduction process. Abed et al. (2018) suggested that although SRB play a key role in AMD remediation, they account for only a small fraction of the total bacteria in the CWs.

Since nitrogen and phosphorus are essential elements for CWs, we also counted the microbial alpha diversity of the control group. We found that after the addition of heavy metals, the richness and diversity of microbial community in CWs decreased to varying degrees. This suggests that heavy metals exert more selective pressure on microorganisms compared to nitrogen and phosphorus, resulting in lower richness and more pronounced dominant populations. The experiments by Zhang et al. (2021e) also demonstrated that the combined pollution of antibiotics and heavy metals had a greater effect on microbial richness than that of a single contaminant, resulting in a decrease in richness values; the higher values of diversity may be due to the variety of pollutants and the need for a wider variety of functional microorganisms to deal with these pollutants.

Overall, the addition of heavy metals causes different degrees of reduction in the alpha diversity of microbial communities in CWs; in particular, the effect on microbial richness is obvious. This is can be seen in Table 5. And the higher the concentration of heavy metals, the greater the impact on alpha diversity.

## Effect of Antibiotics on Alpha Diversity

Antibiotics, due to their mechanism of action, result in lower richness values appear in microbial communities (Tong et al., 2019). However, the median value of the richness of antibiotics is located at a high level. In this case, the increase in microbial richness may be attributed to antibiotics acting as signaling molecules that stimulate the metabolic activity of microorganisms and thereby stimulate the growth of certain microbial species (Li et al., 2021c). With prolonged incubation time, microorganisms were able to gradually adapt to the environment and accumulate, leading to an increase in the richness of the microbial community. This is similar to the effect of heavy metals on microbial richness, but the difference is that the functional microorganisms involved in antibiotic removal can use antibiotics as carbon sources, resulting in a higher microbial richness (Zheng et al., 2021b). Based on the data presented in Table 5, in some studies, after the addition of antibiotics, the richness values were even higher than those in the control group.

Regarding diversity, different antibiotics correspond to different functional microorganisms and therefore easily lead to the formation of dominant population (Yuan et al., 2020). This explains the low values of both diversity indices in the presence of antibiotics.

Overall, antibiotics, like heavy metals, can have a significant impact on microbial alpha diversity, resulting in large differences among the various studies considered here. This reflects the fact that microbial communities may both take longer to remain stable and acquire the corresponding resistance or ability to remove contaminants in the presence of antibiotics or heavy metals (Yu et al., 2020; Zheng et al., 2021b).

## Conclusion and Perspectives

The microbial community, as an important component of CWs, plays a critical role in the removal of pollutants. According to the results of this review, research on microorganisms is gaining increased attention with the advancement of molecular bioanalysis techniques, and studies on microorganisms in CWs have gained considerable importance. This review provided a summary of the functional microorganisms involved in the removal of nitrogen, phosphorus, heavy metals, and antibiotics, the most frequently studied typical pollutants in CWs. This can help researchers to find links between functional microorganisms and pollutants, as well as facilitate the discovery of more relevant functional microorganisms. By summarizing the main functional microorganisms in CWs, we found that the phylum *Proteobacteria* is the dominant one, containing microorganisms with a wide range of functions. In addition, the phyla *Bacteroidetes*, *Actinobacteria*, and *Firmicutes* are also frequently detected in CWs. These functional microorganisms can remove pollutants from CWs by catalyzing chemical reactions, biodegradation, biosorption, and supporting plant growth, etc. The complexity of the microbial community structure and limitations of microbial analysis techniques make it difficult to draw other general conclusions. Regarding the effects of different pollutants on microbial diversity, we found that different microorganisms respond in different ways. When CWs contain high nitrogen and phosphorus levels, functional microorganisms associated with nitrogen and phosphorus removal become dominant in the system, and numerous cross-over phyla or genera of functional microorganisms have been identified. This indicates that research on nitrogen and phosphorous in CWs is advanced and that the removal mechanisms are well understood. In the case of heavy metals or antibiotics, the system can evolve microorganisms adapted to these substances. However, because studies on these two pollutants are scarce, the results cannot be generalized. Overall, heavy metals and high concentrations of nitrogen and phosphorus decrease both microbial richness and diversity in CWs, whereas antibiotics cause large fluctuations in alpha diversity.

Research on the microbial treatment of pollutants in CWs has achieved tremendous breakthroughs and advances with the development of various technologies, but some aspects deserve further investigations:

- Functional microorganisms in CWs should be studied and analyzed more frequently. They play an important role in the removal of pollutants but are often not the dominant microbes. Many studies have focused on the analysis of the overall microbial profile in the system, whereas research on the profile of functional microorganisms in the system is still lacking. In the future, it may be possible to focus on functional microorganisms and to investigate more deeply the composition and diversity of these microorganisms and the influence of different factors on their growth and development.
- Plant-microbe interactions are critical in the removal of contaminants in CWs, and many functional microorganisms associated with the removal of heavy metals, antibiotics, and organic pollution are rhizobacteria or endophytic bacteria isolated from plants. Therefore, further research on plant-associated microorganisms can deepen our understanding of the role of specific microorganisms and plants acting together in the removal of contaminants from CWs.
- There is a lack of studies on microorganisms involved in heavy metal, antibiotics, and some emerging pollutants (such as pesticides, flame retardants, and Polychlorobiphenyls) removal, and often, some specific strains are cultured or isolated to improve the removal efficiency. Therefore, research on these aspects needs to be intensified.
- In the future, more emphasis should be placed on the study of microorganisms at the genetic level, determining their functional enzymes or functional genes. However, such an approach is dependent on the technological advances.

## Author Contributions

JW was responsible for data curation and formal analysis. JW and YL wrote the manuscript. GY contributed to conceptualization, the formal analysis, and visualization. GW, ZZ, PL, YZ, KY, and SW provided feedback on the manuscript. YL supervised, validated, reviewed, and edited the manuscript. All authors contributed to the article and approved the submitted version.

## Funding

This work was supported by the National Natural Science Foundation of China (grant no. 52079010), the Natural Science Foundation of Hunan Province (no. 2021JJ30728), the Scientific Research Fund of Hunan Provincial Education Department (Project Contract No.: 19A032), and the Scientific Research Projects of Ecology and Environment Department of Hunan (no. HBKT-2021012).

## Conflict of Interest

The authors declare that the research was conducted in the absence of any commercial or financial relationships that could be construed as a potential conflict of interest.

## Publisher’s Note

All claims expressed in this article are solely those of the authors and do not necessarily represent those of their affiliated organizations, or those of the publisher, the editors and the reviewers. Any product that may be evaluated in this article, or claim that may be made by its manufacturer, is not guaranteed or endorsed by the publisher.
